# Supplementary material for: Tracking Prostate Carcinogenesis over Time through Urine Proteome Profiling in an Animal Model: An Exploratory Approach
Source: Int J Mol Sci. 2022 Jul 8;23(14):7560. doi: 10.3390/ijms23147560 (PMC9315930; doi:10.3390/ijms23147560)
Supplement: Supplementary file 1 [file ijms-23-07560-s001.zip › Figures S1-S4, Table S1.pdf]

Supplementary Materials

Table S1: Prostate histological lesions and malignancy score for each of the animals whose urine has been analyzed by GeLC-MS/MS (five animals *per* group). When multiple lesions (ML) were observed, the one with higher malignancy was considered.

Animal	Histological lesions	score
Cont1.1	NL	0
Cont1.2	NL	0
Cont1.3	NL	0
Cont1.4	NL	0
Cont1.5	NL	0
PCa1.1	carcinoma	1
PCa1.2	dysplasia	1
PCa1.3	NL	0
PCa1.4	NL	0
PCa1.5	carcinoma (ML)	2
Cont2.1	NL	0
Cont2.2	NL	0
Cont2.3	dysplasia	1
Cont2.4	dysplasia	1
Cont2.5	NL	0
PCa2.1	carcinoma (ML)	2
PCa2.2	carcinoma (ML)	2
PCa2.3	carcinoma (ML)	2
PCa2.4	carcinoma (ML)	2
PCa2.5	carcinoma (ML)	2

Table S2: List of all proteins identified by GeLC-MS/MS.

Table S3: List of proteins identified in the urine of all animals and used for comparative purposes.

Table S4: List of proteins identified in the urine of PCa1 vs.Cont1, PCa2 vs. Cont2, PCa2 vs. Cont2, and Cont2 vs. Cont1 animals. The biological processes and cellular component are present. The p-values were adjusted for multiple-testing. The proteins whose abundance was statistically different between groups were highlighted. Moreover, a comparison between our results and the ones from recent human urinary proteomics data (deposited in PRIDE) was made (“ + “ symbolizes that the result was the same as ours in terms of increasing or decreasing abundance).

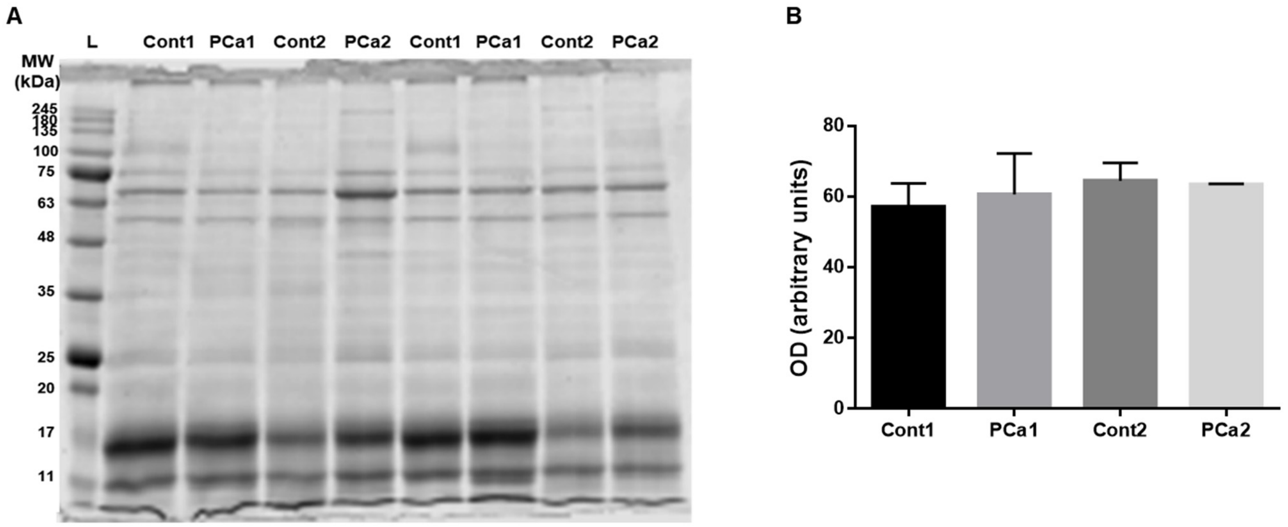

Figure S1: (A) Representative SDS-PAGE of rat urine protein separation for all groups considered and (B) the optical density of total lanes in each group (5 animals per group). The ladder's (L) molecular weights are also displayed.

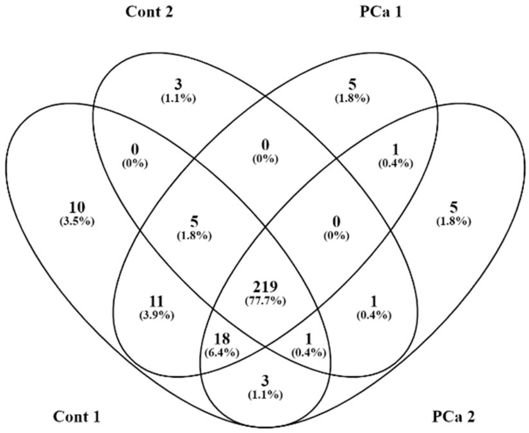

Figure S2: Venn diagram highlighting the distribution of MS identified proteins *per* group.

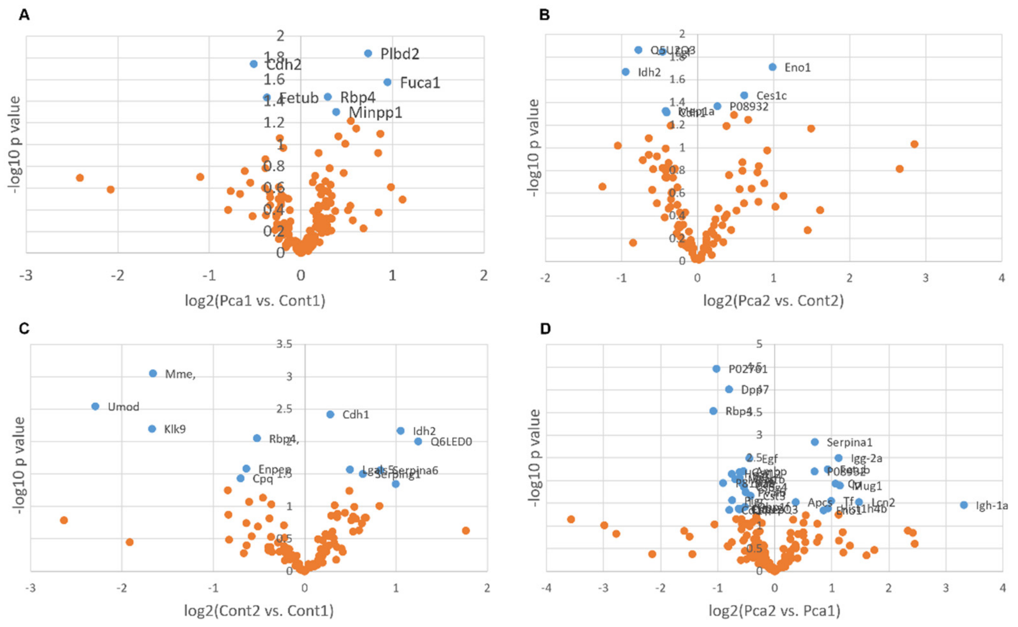

Figure S3: Volcano plots highlighting the up- or down-regulated proteins when comparing the groups (A) PCa1 and Cont1, (B) PCa2 and Cont2, (C) Cont2 and Cont1, and (D) PCa2 and PCa1.

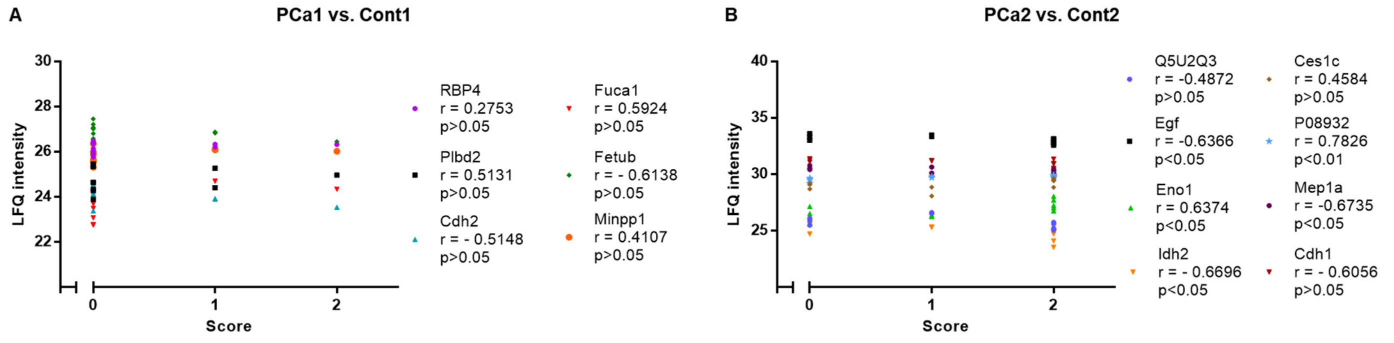

Figure S4: Correlations between proteins abundance (LFQ intensity) in (A) PCa1 vs. Cont1 and (B) PCa2 vs. Cont2 with the prostate histological score.
